# FT-IR Analysis of Structural Changes in Ketoprofen Lysine Salt and KiOil Caused by a Pulsed Magnetic Field

**DOI:** 10.3390/bioengineering9100503

**Published:** 2022-09-24

**Authors:** Salvatore Andrea Pullano, Gianmarco Marcianò, Maria Giovanna Bianco, Giuseppe Oliva, Vincenzo Rania, Cristina Vocca, Erika Cione, Giovambattista De Sarro, Luca Gallelli, Pietro Romeo, Antonio La Gatta, Antonino S. Fiorillo

**Affiliations:** 1 BATS Laboratory, Department of Health Sciences, “Magna Græcia” University of Catanzaro, 88100 Catanzaro, Italy; 2 Clinical Pharmacology and Pharmacovigilance Unit, Department of Health Sciences, “Magna Græcia” University of Catanzaro, Mater Domini Hospital, 88100 Catanzaro, Italy; 3 Department of Surgical and Medical Sciences, “Magna Græcia” University of Catanzaro, 88100 Catanzaro, Italy; 4 Department of Pharmacy, Health and Nutritional Sciences, Department of Excellence 2018–2022, University of Calabria, Ed. Polifunzionale, Arcavacata di Rende, 87036 Rende, Italy; 5 GalaScreen Laboratories, University of Calabria, Ed. Polifunzionale, Arcavacata di Rende, 87036 Rende, Italy; 6 Medifarmagen SRL, University of Catanzaro, 88100 Catanzaro, Italy; 7 FAS@UMG Research Center, Department of Health Sciences, “Magna Græcia” University of Catanzaro, 88100 Catanzaro, Italy; 8 Department of Orthopedics, Istituto di Ricovero E Cura A Carattere Scientifico, Istituto Ortopedico Galeazzi, 20123 Milan, Italy

**Keywords:** magnetic field, diamagnetic pump, FT-IR, structural modification, CRPS, physical therapy

## Abstract

High-intensity, low-frequency magnetic fields (MFs) have been widely used in the treatment of diseases and in drug delivery, even though they could induce structural changes in pharmacological molecules. Morphological changes in ketoprofen and KiOil were investigated through Fourier-transform infrared spectroscopy (FT-IR). Unsupervised principal component analysis was carried out for data clustering. Clinical validation on 22 patients with lower back pain was managed using diamagnetic therapy plus topical ketoprofen or KiOil. The Numerical Rating Scale (NRS) and Short-Form Health Survey 36 (SF-36) were used to evaluate clinical and functional response. Ketoprofen showed clear clustering among samples exposed to MF (4000–650 cm^−1^), and in the narrow frequency band (1675–1475 cm^−1^), results evidenced structural changes which involved other excipients than ketoprofen. KiOil has evidenced structural modifications in the subcomponents of the formulation. Clinical treatment with ketoprofen showed an average NRS of 7.77 ± 2.25 before and an average NRS of 2.45 ± 2.38 after MF treatment. There was a statistically significant reduction in NRS (*p* = 0.003) and in SF-36 (*p* < 0.005). Patients treated with KiOil showed an average NRS of 7.59 ± 2.49 before treatment and an average NRS of 1.90 ± 2.26 after treatment (*p* < 0.005). SF-36 showed statistical significance for all items except limitations due to emotional problems. A high-intensity pulsed magnetic field is an adjunct to topical treatment in patients with localized pain, and the effect of MF does not evidence significant effects on the molecules.

## 1. Introduction

Needleless intradermal drug delivery represents a valid alternative to classic intracutaneous techniques in the treatment of various pathologies [1,2]. To date, in a clinical setting, patient management involves a multidisciplinary approach, meaning joint efforts are indispensable, as in the case of drug treatment and rehabilitation. The latter makes it possible to take charge of the patient at all stages of the disease, improving his or her response to treatment, providing timely access to rehabilitative and supportive therapies, and providing effective management in the event of any disease recurrence [3]. Intradermal pharmacological treatment and rehabilitation have applications for various diseases, such as lower extremity ulcers, chronic wounds, and anatomical and functional recovery of neurological compartments [4,5]. From a physical point of view, the molecules delivered should have specific dermal transport properties and should withstand the action of external physical factors, maintaining their properties (structural and functional integrity) [6]. From a pharmacological point of view, it is important that they offer pharmacotherapeutic action, even at low concentrations [7]. The delivery method has to guarantee high levels of sterility (introducing the molecule of interest into the body in a controlled manner) and safety (procedure performed under controlled conditions) [8,9,10].

Magnetic fields (MFs) have been amply investigated in the literature, evidencing a series of biomagnetic effects at the cellular and organism levels [11,12]. The effects of high-intensity MF include significant variations in the biological activities of various tissues, resulting in positive therapeutic effects both in vivo and in vitro [13]. At the cellular level, electrical repolarization activity of the cell membrane was found, which produced anti-stress action and the regeneration of injured cells. Even though the mechanism has not been fully explained, the literature suggests that MF exposure of cells increases the concentration of opened Na^+^ and Ca^2+^ voltage channels [14]. Furthermore, its action carried out on free radicals, on trace elements, and on substances of the intracellular compartment, contributes to maintaining the homeostatic balance of the cell [15]. Other interesting effects are related to structural changes in DNA and anti-proliferation of tumoral cells [15,16]. In clinical applications, diamagnetotherapy (intensity of hundreds of mT) promotes the acceleration of all reparative phenomena, along with bio-regenerating, anti-inflammatory, anti-edematous, and analgesic actions [17,18]. Studies have evidenced that drug diffusion into a specific human body site can be controllable (preventing/enhancing its diffusion from the target) using MF. One of the concerns is related to its intensity. Even magnetic fields of a few hundreds of mT were reported to be able to influence DNA structure. The literature reports that in some cases, a higher intensity is required (e.g., 50–100 T). These levels cannot be easily generated, and in most cases, they are obtainable under a pulsed regime [19]. At the same time, another important mechanism, which is still not fully analytically described is the role of the MF in the transport of biologically active molecules which can be used as co-adjuvants in the treatment of the above-mentioned diseases. Thus, in addition to treatment, magnetic fields can deliver active molecules through biological barriers (e.g., stratum corneum). The influences of external agents on biological systems and on pharmacological molecules can cause changes in their pharmacological properties (intensity, frequency, etc.). The synthesis of drugs with specific polymorphisms (e.g., carbamazepine, indomethacin, and paracetamol) results in unique properties, and thus external physical factors can affect their biological effects [20].

Infrared spectroscopy is an absorption spectroscopic technique which studies the chemical conformations of molecules. This technique allows one to label an infrared spectrum by plotting the infrared absorption as a function of the wavelength. It is characterized by absorption peaks related to specific functional groups which are part of the structure. In this context, Fourier-transform infrared spectroscopy (FT-IR) is a reliable technique for the characterization of chemical bonds of a molecule. FT-IR provides manifold information, and the interpretation and extraction of useful parameters are sometimes tricky. Attenuated total reflection (ATR) is often associated with FT-IR to analyze liquid samples without preparation techniques. Multivariate classification models are commonly employed to segregate clusters and allow immediate detection of biochemical markers [21]. In chemometrics, due to problems of collinearity and ill-conditioned data, variable reduction with principal component analysis (PCA) is often employed. PCA is one of the most investigated techniques since it reduces the spectral variables into a small dataset with each component orthogonal to each other. Its application is variegated, and different examples can be found in the most recent literature [21,22,23]. The exploratory analysis with PCA on preprocessed spectra represents a simple, reliable, and fast analytical approach.

**Figure 1:**
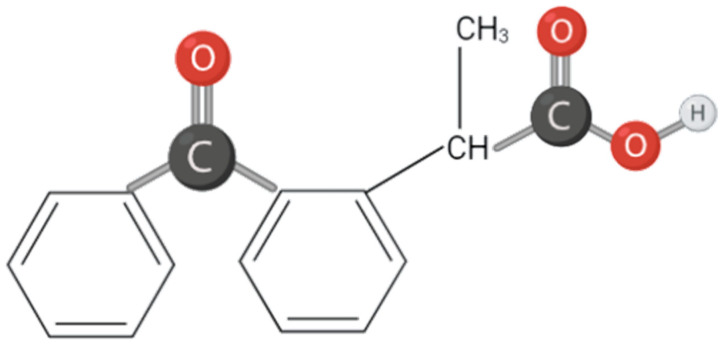
Ketoprofen structure.

In this field, several substances of chemical or natural origin are evaluated for the concomitant transdermal administration with diamagnetic therapy. Ketoprofen (2-(3-benzoylphenyl) propanoic acid), a propionic acid derivative is a non-steroidal anti-inflammatory drug (NSAID). Ketoprofen is an oxo monocarboxylic acid that consists of propionic acid substituted by a 3-benzoylphenyl group at position 2. Ketoprofen is performed on four main functional groups: two aromatic rings separated by a ketone group and a carboxylic acid group [24,25]. The inhibition of prostaglandin synthesis was found since it is able to pass through the blood–brain barrier, with an easier diffusion throughout the central nervous system. Ketoprofen is widely used in several pathologies for pain and inflammation management. However, its side effects (especially in gastrointestinal system and in kidneys) are an important limit for its long-term use [26,27] (Figure 1). 

Arnica exerts anti-inflammatory, anti-microbial, antioxidant and immunomodulatory properties. It may reduce nitric oxide (NO) and Tumor Necrosis Factor α (TNF α) synthesis in macrophages and increase the production of antinflammatory cytokine interleukin-10 (IL-10). Anti-oedemic action was also documented [28]. Widrig et al. documented in 204 patients with hand osteoarthritis that topical arnica had a comparable effect to topical ibuprofen in relieving symptoms [29]. The active components in arnica may reduce nitric oxide (NO) and TNF α synthesis in macrophages and increase the production of anti-inflammatory cytokine interleukin-10 (IL-10). Anti-oedemic action was also documented [28]. Menghini et al. reported that devil’s claw (*Harpagophytum procumbens)* was more effective than placebo in reducing lower back pain and inflammation [30,31,32]. The main components of devil’s claw are iridoid glycosides, such as harpagoside, procumbide and harpagide. However, several other substances such as terpenes, sugars, phytosterols and flavonoids are contained in the plant. Harpagoside seems to act the major role in therapeutic actions. The reduction in inflammatory cytokines, NO and 8-hydroxyguanine (8-OH/Gua) seems to play a role in pain management [30]. Moreover, harpagoside inihibited lipopolysaccharide (LPS) induces the production of COX-2 and NO, acting on the nuclear factor kappa-light-chain-enhancer of activated B cells (NF-κB) [33]. Green tea (one of the most popular drugs in the world) showed antioxidant, anti-inflammatory, and antinociceptive effects through the expression of molecules called catechins [34]. This substance showed efficacy in inflammatory pathologies including rheumatoid arthritis and osteoarthritis [35]. In a randomized clinical trial performed in 44 patients with post-operative pain following third molar surgery, Eshghpour et al. documented a clinical improvement after green tea treatment [36]. Similar evidence was obtained in an open-label study on 50 patients with knee osteoarthritis [37]. Green tea exerts its antinociceptive properties mainly through epigallocatechin-3-gallate (EGCG). It may reduce inflammatory cytokines genes expression. Other catechins may act differently, affecting the functioning of polymorphonuclear leukocytes (PMNs) and assembly of active NADPH-oxidase, scavenging of superoxide anions and inhibition of myeloperoxidase [37]. 

The aim of the study is to determine the effect of a high-intensity low-frequency pulsed MF on the spectroscopic characteristics of topical drugs, specifically on ketoprofen lysine salt (which belongs to the propionic acid derivative family) and KiOil (a patented nutraceutical product) [20]. Since morphological and functional changes in exposed molecules can have a clinical impact if used during a treatment, a clinical study on 22 patients with lower back pain was managed with the same MF therapy plus topical ketoprofen or topical KiOil.

## 2. Materials and Methods

### 2.1. Compounds

Ketoprofen Lysin Salt is a non-steroidal anti-inflammatory drug (NSAID) with anti-inflammatory, antinociceptive, antipyretic effects. It exerts the effects through cyclooxygenase (COX) 1 and 2 inhibition and consequential prostaglandin synthesis blockage [27].

KiOil^®^ is a patented combination of arnica, devil’s claw, green tea, and liposomes created by Medifarmagen SRL a Spin-off of Catanzaro’s University. These substances showed anti-inflammatory and analgesic properties, as described before [28,30,31,32,34]. 

### 2.2. Magnetic Field

The magnetic treatment was performed using diamagnetic equipment (Diamagnetic Pump CTU MEGA 20^®^, Periso SA. Pazzallo-Switzerland). 

Magnetic field can be evaluated at a distance of 2 mm, corresponding to the thickness of the handpiece, in the area adjacent to the coil, using the following relationship:

(1) $$B=\mu_{0}nI/\left(2\pi R\right)$$

where µ_0_ is the vacuum magnetic permeability, n is the number of turns of the winding, I is the current density, and R is the wall thickness. Being a toroidal solenoid, the measured field outside the toroid is negligible. Considering the magnetic field evaluation on the main axis of the solenoid (n = 80 and I equal to 1000 A), the approximate MF is 100.5 mT. Magnetic field at a depth of 2 mm is 88 mT, at a depth of 4.2 cm is 6.3 mT and at a depth of 5.2 cm is about 2.9 mT. The rise time of the field is 2µs the pulse duration is 5 ms (for a period 1 s the energy is 90 J).

**Figure 2:**
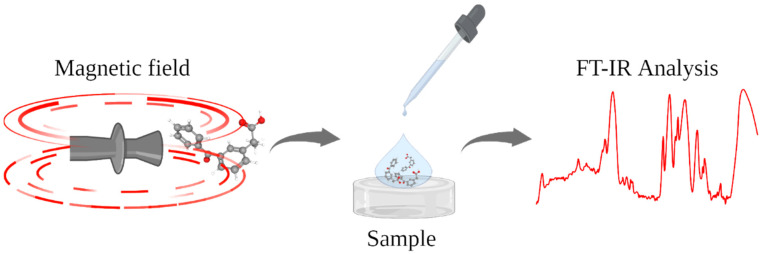
Schematization of the proposed analysis of molecules (i.e., ketoprofen and KiOil) through a diamagnetic pump equipment to generate a controllable MF, and a spectroscopic analysis to investigate its effect.

Investigation performed on ketoprofen lysine salt, and KiOil benzylpenicillin sodium salt was carried out emulating a pulsed magnetic treatment widely used for different pathologies (e.g., low back pain, localized inflammatory process, etc.). The procedure was divided into three steps, the first one performed at a frequency of 1 Hz, a time duration of 10 min, and a pulse energy of 80 J. The second step with a duration of 10 min, a pulse frequency of 3 Hz, and a pulse energy of 90 J, whereas the last step consists of 5 min treatment at a frequency of 5 Hz and a pulse energy of 90 J. A substrate of 10 × 10 × 5 cm composed by polydimethylsiloxane (PDMS) was used as tissue simulating phantom. After the treatment the sample was analyzed in a timeframe no longer than 5 min (Figure 2).

### 2.3. FTIR and Signal Processing

**Figure 3:**
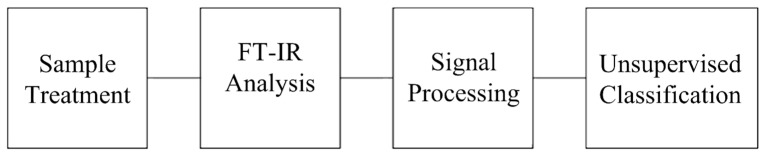
Workflow of sample acquisition and data analysis.

FT–IR ATR (Thermo Scientific NICOLET 6700 FT-IR) was used for the analysis of active molecules (e.g., KiOil, ketoprofen), using attenuated total reflection (ATR) modality. The latter involves the internal reflection of light that interacts inside the sample for few μm allowing molecular profiling. Each spectrum was acquired in the band from 4000 to 650 cm^−1^, averaging 50 acquisitions. Samples were analyzed in wet condition and after being dried at 50 °C under nitrogen flow for 2 min before the analysis. Data preprocessing is used to maximize the signal-to-noise ratio (SNR). This is essential to correctly reduce the effect of physical interference, such as light scattering, different sample thicknesses, different optical paths, and inherent instrument noise. Standardization is also important to reduce the differences between the different systems used. Before any further processing, the spectrum is truncated to the region of interest (e.g., 900–1800 cm^−1^) prior to analysis. This shorter wavenumber region contains the main absorption band of biochemical compounds and suffers only of minor effects of environmental variations (e.g., humidity, temperature). Pre-processing is also aimed at improving the robustness and accuracy of multivariate analysis for increasing the interpretability of the acquired data. Denoising was implemented with Savitizky–Golay (SG) smoothing, which performs filtering based on least squares fit of each set of data using a second-degree polynomial with a 9-point window. Artifacts in the scattering of the incident light were reduced by implementing extended multiplicative scatter correction (EMSC), improving spectra normalization (9-point second derivative, respect to a unity vector). Principal component analysis was used as unsupervised clustering technique for data and pattern recognition (Figure 3) [22]. PCA decomposes the original spectral matrix X into scores (S), loadings (L), and residuals (R) as follows:

X = SL^s^ + R (2)

S evaluates clustering patterns among the samples and represent the variance on sample direction; L detects the variables that evidence the highest importance for the observed pattern on the scores and represent the variance on wavenumber direction; R points out eventual errors. PCA is useful to reduce dataset dimension in a small number of principal components (PCs) that cover most of the spectral data variance. The first PC represents the most part of data variance, followed by the second orthogonal PC, etc. [23].

### 2.4. Study Design

We conducted a pilot single-center open-label randomized clinical study between 1 June 2021, and 30 March 2022 in patients with lumbar pain referred to our ambulatory of Pain Medicine of the Operative Unit of Pharmacology and Pharmacovigilance of the University of Catanzaro. The study, approved by the Ethics Committee of Calabria Centro (number 15/2022), was carried out according to the Good Clinical Practice guidelines and with the ethical principles of the Declaration of Helsinki. Before the beginning of the study, all participants signed written informed consent.

### 2.5. Experimental Study

Patients with a clinical and instrumental (Magnetic Resonance imaging) diagnosis were enrolled and randomized to receive a topical treatment with topical NSAIDs (ketoprofen) (Group 1) or fixed compound (KiOil) (Group 2). Patients enrolled were male or female above the age of 18 with a diagnosis of chronic back pain (>3 months). Excluded patients included those who with pacemakers, prothesis or other implanted metallic devices, and with a diagnosis of cancer. At the time of enrollment (T_0_), and 2 months later (T_1_: end of treatment), the physician blinded to the group of the study evaluated the NRS (Numerical Rating Scale) in each treatment session and evaluated patients’ functionality through the Short Form Health Survey 36 (SF-36) questionnaire. Student’s paired *t*-test was performed with GraphPad. Diamagnetic therapy protocols used, according to the diamagnetic equipment (Diamagnetic Pump CTU MEGA 20^®^-Periso SA. Pazzallo, Switzerland) manual, are summarized in Table 1.

## 3. Results

**Figure 4:**
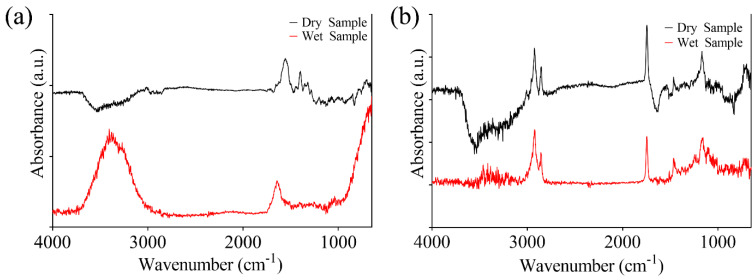
Comparison between spectra obtained from wet and dry sample of ketoprofen lysine salt and excipients (**a**), and KiOil (**b**).

Infrared spectra of wet and dried samples (at 50 °C under nitrogen flow for 2 min), were firstly analyzed. In Figure 4a,b, the spectrum of ketoprofen lysin salt in gel is reported. 

**Figure 5:**
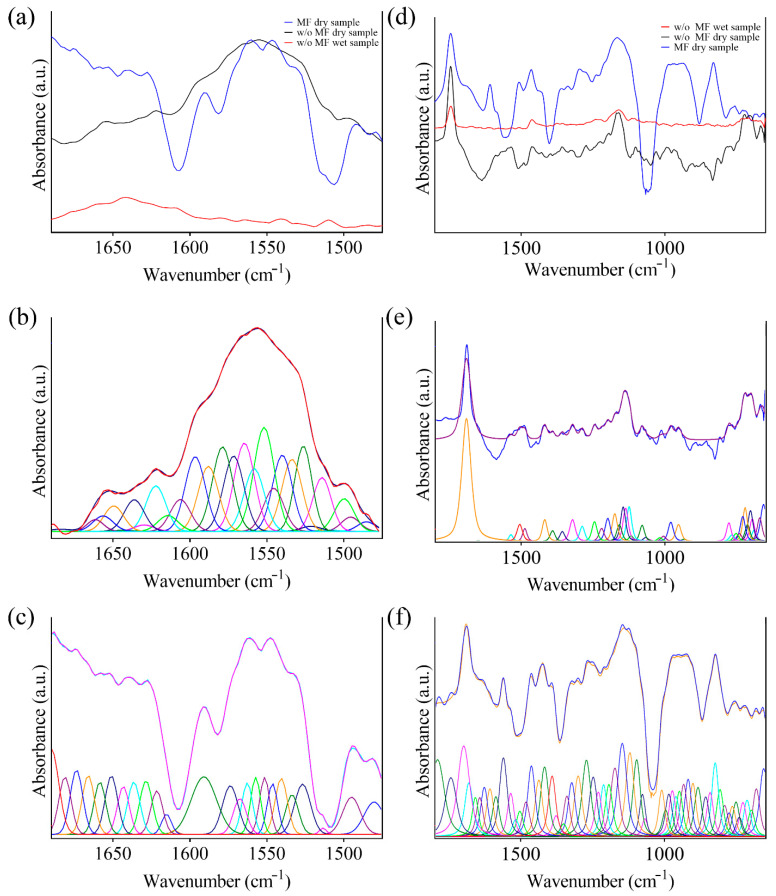
FT-IR spectra of (**a**) ketoprofen dried sample before and after MF treatment and of wet sample non exposed to magnetic field. Curve-fitting of (**b**) the ketoprofen control dried spectrum and (**c**) dried spectrum after MF treatment. (**d**) KiOil dried sample before and after MF treatment and of wet sample non exposed to magnetic field. Curve-fitting of (**e**) the KiOil control dried spectrum and (**c**) dried spectrum after MF treatment (**f**).

The gel is based on ketoprofen lysine salt and other excipients (e.g., carboxypolymethylene, triethanolamine, ethyl alcohol, polysorbate 80, methyl para-hydroxybenzoate, purified water, etc.). The wet components of the formulation provide undesirable effect on the spectrum, creating a shadowing of molecular specific absorption bands. Figure 4a evidenced a strong absorption band between 4000 cm^−1^ and 3000 cm^−1^ which is due to excipients, such as water. Previous investigations performed on biological samples evidenced how a proper removal of these components does not cause statistically significant differences among samples [22,23]. Moreover, drying of sample results in a less contribution of the interfering molecules, evidencing the onset of strong absorption band which were not previously highlighted. The latter are mainly localized in the fingerprint region (less than 2000 cm^−1^). Dehydrated samples can be further analyzed to investigate the chemical/physical state of the drug in the considered formulation. The literature evidenced how pure ketoprofen is characterized by two absorption peaks at about 1655 cm^−1^ and 1697 cm^−1^, which are mainly due to C=O stretching in the dimeric carboxyl acid and ketonic group [38]. The considered formulation includes L-Lysine, that has been shown to cause a weaker absorption band since part of the molecule is not in the crystalline form. This results in a weaker C=O stretching absorption band [38,39]. The presence of different excipients gives rise to other absorption band such as that of Methyl 2-hydroxy benzoate (1676 cm^−1^ in the wet sample, shifted up to1588 cm^−1^ in the dry sample). Thus, the overall spectra can be heterogeneous and multiple interference can cause the onset of new absorption bands. The same analysis performed on KiOil which is a nutraceutical product evidenced a much lower contribution to the shadowing effect by interfering molecules. In this case, the absence of water inside the formulation is beneficial for FT-IR analysis. Comprising a mixture of different components (arnica, devil’s claw, green tea, liposomes), FT-IR spectra results were composed of related absorption bands. For example, arnica is characterized by strong adsorption bands around 1740 cm^−1^ and 1160 cm^−1^ caused Carbonyl stretching. Green tea is characterized by a strong absorption band around 1640 cm^−1^ (carboxylate), and other lower peaks in the bands from 1200 down to 800 cm^−1^, whereas pure Devil’s claw spectra evidenced absorption bands correlated to ether linkages (1145, 1620 cm^−1^), and phenolic OH groups (around 3000 cm^−1^) present in the Harpagoside [40,41,42]. The study of the effect of pulsed magnetic field was carried out on 15 identical drug formulations of ketoprofen lysine salt gel and KiOil. The overall exposure time of the three steps was 25 min over a PDMS background, followed by FT-IR/ATR analysis within 5 min. Two control samples were considered, one obtained from the not exposed wet formulation and the other one from the not exposed dry sample. As previously evidenced, most of the structural/molecular data of the sample are in the fingerprint region (less than 2000 cm^−1^), more in particular down to 800/1000 cm^−1^. Figure 5a showed the spectra of ketoprofen lysine salt control samples and as a result of low frequency pulsed MF action. Peak resolving was performed by curve-fitting of the FT-IR spectra in each selected region for dried samples using a Voigt function maintaining fixed parameters (i.e., baseline, full width half height) during fitting for ketoprofen (Figure 5b,c) and KiOil (Figure 5e,f). The specific peak frequency of fitted spectra reported in Figure 5 are summarized in Table 2. In this case, for ketoprofen, FT-IR analysis provides evidence of a change in this pattern, which means that some of the components of the drug structurally changed after the interaction with magnetic fields. KiOil seems less affected by structural change in the frequency band considered, since most of the vibrational peaks are maintained. 

**Figure 6:**
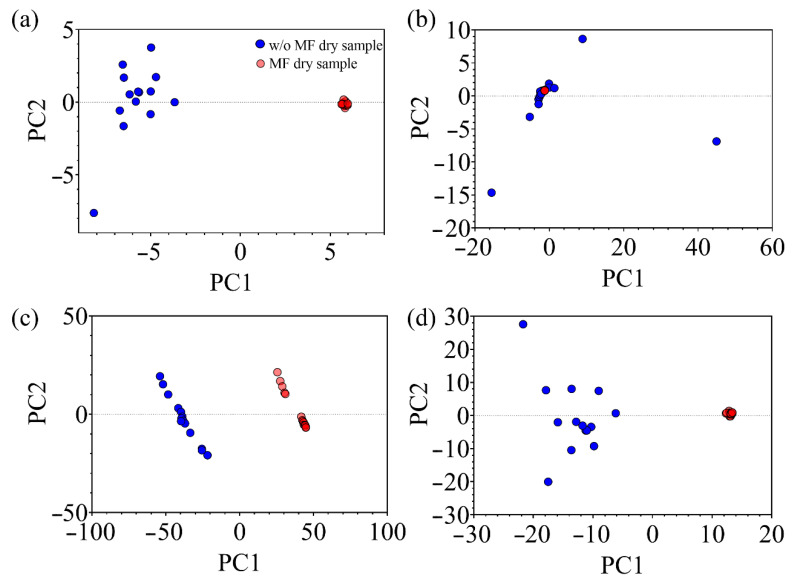
(**a**) PCA score plot of ketoprofen in the range 4000–650 cm^−1^ (**b**), and in the range 1675–1475 cm^−1^ (**c**). PCA score plot of KiOil in the range 4000–650 cm^−1^ (**d**), and in the range 1800–650 cm^−1^.

Since it is tricky to monitor all the absorption bands in FT-IR analysis and their cross-interaction inside the different formulation, PCA was applied to the spectra in order to reduce variability and dimensionality of the data. In Figure 6 is reported a two-dimensional plot (PC1 vs. PC2) on each formulation performed on the overall spectrum and in the specific band reported in Figure 5 (1675–1475 cm^−1^ for ketoprofen lysine salt and 1800–600 cm^−1^ for KiOil). In Figure 6a, the analysis over the overall spectra for ketoprofen lysine salt is reported using PC1 vs. PC 2, which accounted for over 90% of the cumulative total variance. Here, a clear pattern between samples exposed to MF respect to those not exposed. Both classes of samples result clustered, even though a greater dispersion can be evidenced in not exposed samples. The same analysis was performed in a narrow wavenumber band (1675–1475 cm^−1^) which contains specific features of the ketoprofen together with lysine salt (as showed in Figure 6b). The latter result could provide evidence that even though the formulation experience structural changes in some components, it does not affect the ketoprofen lysin salt molecules. KiOil has instead shown consistent results on the overall spectra (Figure 6c) and in the range 1800–650 cm^−1^), evidencing that structural modifications in the nutraceutical product affect the formulation also in the fingerprint region (Figure 6d). Being composed of different nutraceutical components, this result should be taken into consideration when using the formulation.

### 3.1. Clinical Treatment with Ketoprofen 

Diamagnetic therapy was administered to 11 patients in addition to ketoprofen with previous pharmacologic treatment and with low back pain. Cohort characteristics are summarized in Table 3.

Patients’ ages were quite high, at 64.1 ± 8.46 years. Females represented the majority of this group, with eight patients (72.7%). Hypertension and mild cardiovascular affections; osteoporosis/osteopenia, dysthyroidism and hyperlipidaemia were the most frequent comorbidities. All patients were previously treated with at least one pain controlling drug. The totality of the group (eleven patients) received NSAIDs (Table 3).

A mean of 7.77 ± 3.09 sessions with PMFs were performed. During this period, nine patients (81.8%) consumed pain drugs. In total, six patients (54.6%) continued NSAID consumption during the diamagnetic therapy period: this choice was autonomous since we tried to reduce NSAID consumption. Interestingly, in their last session, six patients (54.6%) were still consuming a drug, evidencing a reduction compared with the beginning of the treatment. The Medium Numerical Rating Scale before treatment was 7.77 ± 2.25, whereas it was 2.45 ± 2.38 after treatment. This was statistically significant (*p* = 0.003, < 0.05). SF-36 questionnaire data are available in Table 4.

The SF-36 questionnaire is focused on evaluating pain level, physical and psychological functioning. It is composed of nine items, evaluated on a scale from 0 to 100. Diamagnetic therapy plus ketoprofen reached the statistical significancy in improving all the items comparing SF-36 before and after treatment. In summary, all patients showed important benefits in pain control and in all the functional items of the SF-36 questionnaire.

### 3.2. Clinical Treatment with KiOil 

Diamagnetic therapy was administered to 11 patients, in addition to KiOil with previous pharmacologic treatment and with low back pain. Court characteristics are summarised in Table 5.

Patients age was 58.63 ± 14.71. Females represented the majority of this group (eight patients, 72.7%). Hypertension, hyperlipidemia, gastroenterologic pathologies, obesity, and other cardiovascular illnesses were the most frequent comorbidities. Ten (90.90%) patients were previously treated with at least one pain-controlling drug. A medium number of 7.27 ± 1.48 sessions with PEMFs therapy was performed. During this period, ten patients (90.90%) consumed pain drugs, according to our team, or on their own (Table 6). In their last session, six patients (54.54%) were still consuming a drug, evidencing a reduction compared with the beginning of the treatment. The Medium Numerical Rating Scale before treatment was 7.59 ± 2.49, whereas it was 1.90 ± 2.26 after treatment. It was statistically significant (*p* = 0.0002, < 0.05). SF-36 questionnaire data are available in Table 6. In summary, all patients showed important benefits in pain control and in most of the items of SF-36 questionnaire, except for limitations due to emotional problems, and was near to statistical significance for social functioning and energy fatigue. 

## 4. Discussion 

A standardized protocol has been defined to ensure reproducibility of data during single or multiple repeated analytical sessions. PCA is one of the most popular machine learning techniques for unsupervised clustering. Since our work data frame has a small sample size, PCA results are useful for exploratory analysis, evincing two subgroups covering the most part of variance. The used framework applied for preprocessing and clustering represents an initial step. Further studies aim to increase population size in order to apply a supervised classification and validate the diamagnetic therapy effects. Results obtained through an unsupervised classification technique evidenced not significant structural changes in the ketoprofen respect to KiOil. However, this cannot be suddenly considered as a negative effect since, in some cases, literature evidenced magnetic field have enhancing pharmacological effect. In any case, it is necessary to provide clinical evidence if these changes have beneficial effects or not.

Diamagnetic therapy is based on PEMFs. In a randomised pilot trial by Vicenzino et al., PEMFs were used to transdermally deliver glucosamine, chondroitin and hyaluronic acid in a court of 114 male individuals with knee injuries. The effect of this therapy was compared with NSAID gel application alone. The PEMF group had superior results in pain control [43]. As reported in the literature, PEMFs have been used or experimented with in several clinical conditions, including neuropathic pain [44], ulcer [45], complex regional syndrome [46], epicondylitis [47], and fractures [48]. Concerning our study setting, a recent systematic review by Sun et al. [49] evidenced benefits in pain control from PEMFs treatment in 618 individuals, but not significant improvements in functionality. 

In other pathologies, a RCT by Osti et al., showed short term benefits on shoulder pain (after rotator cuff repair) in a 66 patients court repair. However, these effects in pain control and functionality disappeared at a two-year follow-up [50]. The trial by Binder and colleagues on 29 patients with rotator cuff tendinitis showed improvements in 24 of the 29 subjects treated with PEMFs [51]. Other trials did not reach statistical significance [52,53].

Few information is available on ankle pain. All of these sets of data allow us to highlight an important consideration: pulsed magnetic-field therapy effects may also weaken as time goes by, and functionality improvements are not visible in all subjects. However, parameters, device, and time of treatment varied greatly in all these studies. On the other hand, given the characteristics of diamagnethotherapy of selective interaction with biological tissues, it is important to consider this therapy as a drug finding the proper dosage for each patient and the right protocol basis for the single pathology [54]. 

The efficacy of this therapy may be due to many different mechanisms. Modulation on neuropathic pain component. An animal model by Coksun et al. showed that low-frequency PEMFs are capable to modulate ionic channels in rats. Sodium channels NaV_1.8_ and NaV_1.9_, down-regulated in painful conditions, were re-regulated after PEMF treatment [55]. In an in vitro study by Ahmed et al., the action of PEMFs on depolarization and sodium/potassium channels was described [56]. 

Moreover, PEMFs modulate several ligand binding and receptor activity. They act as antagonist of A2A and A3 adenosin receptors, reducing the release of pro-inflammatory cytokines, the expression of NFκB and of prostaglandin E2 (PGE2). Furthermore, they seem to modulate bone formation [57]. Another important effect is related to increased angiogenesis, related to vascular endothelial growth factor (VEGF), angiopoietin 2 (Ang-2), fibroblast growth factor (FGF) 2 and other targets involved in angiogenesis, as shown by Peng and colleagues in an animal model [58]. Diamagnetic therapy movement of liquids protocol may favor fluid drainage, tissue stimulation, nutrient and metabolite transport, cellular homeostasis. These effects are possible according to diamagnetic therapy action on water and intracellular/extracellular environment [46,59,60]. Our study evidenced an important benefit in patients. It is noteworthy that at treatment ending the percentage of people consuming a drug for pain management lowered (from 81.81% to 54.54% in ketoprofen group and from 90.90% to 54.54% in the KiOil group). Even if some patients did not show a NRS reduction compared with the first session, they were able to reduce drug dosage or improve their functionality. This is very important since drugs for pain management (especially opioids and NSAIDs) are characterized by severe long-term adverse events [61,62].

Topical treatment in addition to diamagnetic therapy seems to be a safe approach in reducing drug adverse events. Despite the possibility of local reactions and a small number of systemic reactions, topical analgesics are sharply safer [63].

SF-36 questionnaire items showed important results. Significant improvements of all items were registered in ketoprofen group. In KiOil group all the items, except energy/fatigue, limitations due to emotional problems and social functioning. This fact should be related to patients baseline high capacity in social and emotional sphere.

Finally, confounding factors may have affected SF-36 results: one patient have rheumatologic comorbidities, since pain level is also influenced by the control of this pathology [64]. Otherwise, psychiatric comorbidity could have affected emotional sphere questions [65]. In general, the presence of other clinical conditions may have influenced patients’ answers on health status and physical activity.

## 5. Conclusions

In this study, we presented a technique for drug analysis of molecules exposed to pulsed high-intensity low-frequency magnetic field using FT-IR ATR and multivariate classification algorithm. The first part of the work was focused on the standardization of a protocol for the analysis of drug molecules of pharmacological interest exposed to MF. Diamagnetic therapy is a useful add-on therapy to manage chronic and acute pain. Millions of patients suffer from pain. Pain is a real syndrome capable of affecting physical and psychological components of individuals. Although PEMFs have been well known for a long time, the absence of specific guidelines, of large RCT and the lack of homogeneity in devices are still important limits that did not allow us to use this therapeutic option in a rigorous way. Our study showed that this therapy is capable to reduce pain and improve physical functioning in certain patients, in addition to pharmacologic treatment. In particular, our analysis on 22 patients with chronic lower back pain showed an important improvement on pain control and functionality. These subjects benefited from the concomitant transdermal administration of topical substances, vehiculated by diamagnetic therapy. Ketoprofen and KiOil are only two of the possible products that may be administered in this clinical context. Diamagnetic therapy may offer a safe approach to manage several chronic pain conditions including shoulder, low back, knee, cervical, and ankle pain of different causes and origins. This approach has a key advantage: the possibility of reducing the number and the dosage of drugs of used for pain management. In fact, these compounds are characterized often by several adverse events and the necessity of chronic consumption. However, our study has an important limitation in the number of patients examined. This study may be the basis for larger RCT dedicated to specific pathologies, and scenarios have been used to assess real-life effectiveness, the most responsive patients, and long-term effects.

## Figures and Tables

**Table 1:** Magnetic field protocol.

Protocol Name	Patients	Parameters
Chronic low back pain	n = 22	Pain control (10 min): 5 Hz, 70 J Endogenous biostimulation (10 min): power 4, skeletal muscle Movement of liquids (5 min): Intracellular 80, extracellular 60

**Table 2:** Wavenumber obtained from curve fitting analysis of the FTIR spectrum.

Ketoprofen				KiOil			
No MF treatment Wavenumber (cm^−1^)		No MF treatment Wavenumber (cm^−1^)		No MF treatment Wavenumber (cm^−1^)		No MF treatment Wavenumber (cm^−1^)	
1480.62 1493.85 1499.32 1506.88 1512.65 1520.54 1525.72 1532.73 1538.24 1544.56 1550.67 1556.67 1564.00 1570.59	1578.08 1587.22 1596.15 1604.70 1613.88 1622.02 1629.21 1635.86 1643.09 1649.49 1655.99 1661.15 1671.53 1690.74	1478.05 1492.89 1501.57 1505.81 1511.88 1525.26 1532.20 1539.31 1545.11 1550.33 1556.24 1561.80 1566.75 1573.07	1590.51 1610.15 1615.21 1621.62 1628.74 1636.79 1643.72 1651.59 1659.18 1666.85 1674.64 1682.25 1691.26	813.95 831.84 850.43 866.69 894.65 913.48 930.98 947.37 962.93 975.85 988.77 1001.68 1013.41 1027.63 1038.15 1054.25 1060.68 1068.51 1089.40 1098.43 1119.60 1144.56 1172.38 1199.40 1221.32 1240.90	1259.83 1278.82 1303.73 1334.04 1357.59 1375.39 1387.70 1396.35 1415.01 1429.54 1457.17 1478.19 1505.45 1525.28 1547.70 1563.15 1581.13 1608.12 1635.53 1657.24 1678.04 1694.91 1710.84 1734.64 1753.47	755.69 770.81 782.00 797.49 815.80 835.68 844.22 859.77 872.34 889.91 906.33 927.07 946.95 967.22 995.53 1022.77 1036.04 1060.81 1075.40 1087.87 1100.83 1111.32 1121.79	1130.01 1141.47 1148.50 1161.34 1170.47 1184.63 1201.17 1226.97 1249.74 1275.81 1320.99 1356.49 1394.12 1429.87 1458.59 1487.75 1531.80 1550.50 1582.85 1627.62 1660.41 1703.38 1746.09

**Table 3:** Characteristics of the study population enrolled for topical treatment of ketoprofen.

Total (n = 11)	
Age, years	64.09 ± 8.45
Years of illness	4.18 ± 4.62
Females, n (%)	8 (72.72)
Smokers or ever smokers, n (%)	3 (27.27)
Diagnosis, n (%)	
Low back pain	11 (100%)
Comorbidities; n (%)	
Cardiovascular illness	5 (45.45)
Diabetes	1 (9.09)
Dysthyroidism	3 (27.27)
Gastroenterologic	2 (18.18)
Hyperlypidaemia	3 (27.27)
Hypertension	7 (63.63)
Neurologic	2 (18.18)
Obesity	2 (18.18)
Osteoporosis/osteopenia	3 (27.27)
Psychiatric	2 (18.18)
Renal	1 (9.09)
Rheumatologic	1 (9.09)
Urologic	1 (9.09)
Other	2 (18.18)
Previous pain treatments, n (%)	11 (100)
Corticosteroids	2 (18.18)
Myorelaxants	2 (18.18)
NSAIDs	11 (100)
Opioids	3 (27.27)
Paracetamol	4 (36.36)
Pregabalin	2 (18.18)
Other	2 (18.18)
Concomitant pain treatments, n (%)	9 (81.81)
Duloxetine	1 (9.09)
L-acetylcarnitine	3 (27.27)
Myorelaxants	2 (18.18)
NSAIDs	6 (54.54)
Opioids	3 (27.27)
Paracetamol	4 (36.36)
Pregabalin	1 (9.09)
Medium number of sessions	7.77 ± 3.09
NRS before treatment	7.77 ± 2.25
NRS after treatment	2.45 ± 2.38
Pharmacologic treatment at last session, n (%)	6 (54.54)

NSAIDs, Non-steroidal anti-inflammatory drugs.

**Table 4:** Ketoprofen SF-36 questionnaire results.

SF-36 Item	First Session	Last Session	Statistical Significancy
Physical functioning Limitations of physical health Limitations emotional problems	44.09 ± 20.59	69.09 ± 17.86	*p* < 0.0001
	20.45 ± 31.26	56.82 ± 27.59	*p* = 0.0039
	72.73 ± 38.92	93.93 ± 20.11	*p* = 0.0456
Energy/fatigue Emotional well being	47.27 ± 18.35	57.73 ± 16.49	*p* = 0.0028
	64.36 ± 15.95	72.00 ± 14.75	*p* = 0.0032
Social functioning Pain General health Health change	81.81 ± 17.99	90.90 ± 11.30	*p* = 0.0119
	32.04 ± 17.05	65.90 ± 19.04	*p* < 0.0001
	38.18 ± 28.42	51.36 ± 18.04	*p* = 0.0047
	29.55 ± 21.85	72.73 ± 23.60	*p* < 0.0001

**Table 5:** Characteristics of the study population enrolled for topical treatment of KiOil.

Total (n = 11)	
Age, years	58.63 ± 14.71
Years of illness	2.18 ± 1.72
Females, n (%)	8 (72.72)
Smokers or ever smokers, n (%)	4 (36.36)
Diagnosis, n (%)	
Low back pain	11 (100)
Comorbidities; n (%)	
Cardiovascular illness	3 (27.27)
Diabetes	2 (18.18)
Dysthyroidism	2 (18.18)
Gastroenterological	3 (27.27)
Hyperlipidaemia	4 (36.36)
Hypertension	5 (45.45)
Neurologic	2 (18.18)
Obesity	3 (27.27)
Osteoporosis/osteopenia	2 (18.18)
Pneumological	1 (9.09)
Renal	1 (9.09)
Urologic	2 (18.18)
Other	5 (45.45)
Previous pain treatments, n (%)	10 (90.90)
Corticosteroids	2 (18.18)
L-acetylcarnitine	3 (27.27)
Myorelaxants	2 (18.18)
NSAIDs	10 (90.90)
Opioids	4 (36.36)
Paracetamol	2 (18.18)
Pregabalin	2 (18.18)
Other	3 (27.27)
Concomitant pain treatments, n (%)	10 (90.90)
Duloxetine	3 (27.27)
L-acetylcarnitine	3 (27.27)
Myorelaxants	6 (54.54)
NSAIDs	6 (54.54)
Opioids	6 (54.54)
Paracetamol	6 (54.54)
Pregabalin	1 (9.09)
Steroids	3 (27.27)
Other	4 (36.36)
Medium number of sessions	7.27 ± 1.48
NRS before treatment	7.59 ± 2.49
NRS after treatment	1.90 ± 2.26
Pharmacologic treatment at last session, n (%)	6 (54.54)

NSAIDs, non-steroidal anti-inflammatory drugs.

**Table 6:** KiOil group SF-36 questionnaire results.

SF-36 Item	First Session	Last Session	Statistical Significancy
Physical functioning Limitations of physical health Limitations emotional problems	55.45 ± 19.81	75.91 ± 18.41	*p* = 0.0017
	31.82 ± 31.80	68.18 ± 29.77	*p* = 0.0039
	90.90 ± 21.56	96.97 ± 10.04	*p* = 0.1669
Energy/fatigue Emotional well being	58.18 ± 9.82	62.73 ± 9.32	*p* = 0.0531
	60.36 ± 8.66	65.82 ± 7.01	*p* = 0.0024
Social functioning Pain General health Health change	84.09 ± 17.75	94.31 ± 6.52	*p* = 0.0552
	37.04 ± 17.74	70.90 ± 10.91	*p* < 0.0001
	28.18 ± 13.28	42.73 ± 14.72	*p* = 0.0012
	18.18 ± 19.66	84.09 ± 12.61	*p* < 0.0001

## Data Availability

Not applicable.
